# Proportion and factors of death among preterm neonates admitted in University of Gondar comprehensive specialized hospital neonatal intensive care unit, Northwest Ethiopia

**DOI:** 10.1186/s13104-018-3970-9

**Published:** 2018-12-06

**Authors:** Ayenew Engida Yismaw, Abebe Ayinalem Tarekegn

**Affiliations:** 1 School of Midwifery, College of Medicine and Health Science, University of Gondar, Gondar, Ethiopia; 2 Department of Health Economics, Institute of Public Health, College of Medicine and Health Sciences, University of Gondar, Gondar, Ethiopia

**Keywords:** Ethiopia, Preterm, Neonate, Death, Neonatal, Intensive care unit

## Abstract

**Objective:**

Neonatal mortality accounts for 43% of under-five child mortality in Ethiopia where preterm is the second leading cause of neonatal death and steadily increased in low-income countries. Therefore, assessing the proportion of death and associated factors among preterm neonates has a paramount importance in designing an effective strategy to intervene and achieve sustainable development goal.

**Results:**

In this study proportion of preterm neonatal death in this study was 28.8% [95% CI (25.1, 32.9)]. Complications during index pregnancy [AOR = 1.92, 95% CI (1.09, 3.38)], gestational age [AOR = 0.78, 95% CI (0.69, 0.91)], small for gestational age [AOR = 2.42, 95% CI (1.33, 4.38)], APGAR score at birth < 7 [AOR = 2.39, 95% CI (1.34, 4.27)], hyaline membrane disease [AOR = 5.15, 95% CI (2.83, 9.36)], neonatal respiratory distress at admission [AOR = 1.93, 95% CI (1.13, 3.31)], presence of jaundice [AOR = (3.39, 95% CI (1.90, 6.05)], received kangaroo mother care [AOR = 0.13, 95% CI (0.05, 0.35)], and hypoglycemia at admission [AOR = 3.86, 95% CI (2.12, 7.06)] were statistically significant. The proportion of preterm neonatal death was high. Ministry of health and responsible organizations should give special attention for preterm neonates to prevent life-threatening complications.

## Introduction

In the globe, approximately 3.1 million and 2.9 million neonatal deaths were reported in 2010 and 2014, respectively, which accountings 40% of the under 5 mortality. Despite a steady decline of neonatal mortality in African countries, it is not satisfactory [1, 2]. Globally, 3 in 4 neonatal deaths were caused by preterm birth complications which accounts for 35% of all neonatal deaths [3]. Infection (36%), preterm birth (28%) and birth asphyxia (23%) are the most common causes of neonatal mortality in the world [4–6].

Preterm (PT), a birth before 37 completed weeks of gestation, is the most frequent cause of neonatal death and the second leading cause of both neonatal and under-five mortality with multiple short and long-term health threats worldwide [7]. Lack of immunologic competence increases the risk of preterm infants for multiple infectious processes which may lead to long-term neurodevelopmental disorders and chronic lung disease [8].

Ethiopian Demographic and Health Surveys reported that neonatal death was increased from 32% in 2005 to 43% in the 2016 and according to United Nations Children’s Fund (UNICEF) report, preterm birth which accounts 23% was believed to be a major and direct cause of neonatal death in Ethiopia [9, 10]. Causal factors linked to preterm birth are medical conditions of the mother or fetus, genetic influences, environmental exposure, infertility treatments, behavioral and socio-economic factors, medically indicated preterm delivery as well as iatrogenic prematurity [11, 12].

Studies reported that preterm neonatal mortality ranged from 15 to 36% [1, 13, 14]. Reports from the worlds’ Low to Middle-income countries showed that 34 to 40% of neonatal mortality were contributed by preterm [5, 15–17]. Hospital-based studies in Africa reported that preterm neonatal mortality accounted for about 15.7 to 29.6% [18, 19]. Studies in Ethiopia reported that preterm neonatal mortality rate ranges from 18% up to more than 40% [20–26].

Different studies conducted so far showed that rural residency [18, 24], maternal age [27, 28], place of birth [20, 29]. Obstetric risk factors were not having ANC [15–17], being prim para [4, 24], having any pregnancy complications [20, 29], Labour and delivery complications [15, 19, 23], having previous bad obstetric history [16, 23] were risk factors for preterm neonatal death.

Neonatal related risk factors include being male sex [15–17, 30], low birth weight at birth [15, 24], gestational age (GA) at birth and neonatal congenital malformations [18, 23], neonatal clinical problems like respiratory distress syndrome (RDS), perinatal asphyxia (PNA), hyaline membrane disease (HMD), jaundice, hypoglycemia, hypothermia and neonatal sepsis [6, 23, 26, 31], timely initiation of breastfeeding upon birth and kangaroo mother care (KMC) were reported as factors of preterm neonatal death [26, 28].

Even if premature birth is not an acute disease, it is one of the major causes of infants’ death and preterm birth continues to be significant public health problem by increasing the average cost of medical care for a premature and low birth-weight baby for the first year of life for developing country like Ethiopia. These high medical expenses could burden both the parents, families as well as the community. Therefore, this is the dual agenda to prevent preterm birth and reduce neonatal death which requires a comprehensive research strategy to end the preventable deaths of newborns and under-five children.

## Main text

### Methodology

#### Study design and setting

Retrospective cross-sectional study was conducted to assess the proportion of death and its associated factors among preterm neonates admitted in NICU from January 2016 to March 2018 in University of Gondar comprehensive specialized hospital neonatal intensive care unit. The hospital is one of the largest teaching hospital found in Amhara region providing tertiary level care for more than seven million people in the North West part of the country.

#### Sample size and sampling procedure

All preterm neonates admitted to neonatal intensive care unit at the University of Gondar comprehensive specialized hospital. Thus, preterm neonates admitted to NICU with a gestational age of less than 37 completed weeks were source population and all preterm neonates who were admitted to neonatal intensive care unit (NICU) at University of Gondar comprehensive specialized hospital from January 2016 to March 2018 were the study population. A total of 516 preterm neonates admitted in NICU were included in the study.

#### Data collection method and instruments

Medical records were reviewed and preterm neonatal cards were identified by their medical registration/card number. Then data were extracted using structured and pretested data extraction checklist prepared in English from HMIS registration format and patients’ card. Trained midwife professionals had collected the data.

#### Data quality assurance and analysis

Data were entered into EPI info version 7 and imported to Stata version 14 statistical software for further analysis. Descriptive statistical data analysis was done and followed by bivariate and multivariable analysis in order to see the statistical association between the outcome and explanatory variables. Variables which showed significant association in the bivariate analysis were entered into multivariable logistic regression. The degree of association was assessed using odds ratio with 95% confidence interval and P-value < 0.05 were taken as statically significant.

### Results

#### Socio-demographic and obstetric characteristics of mothers

In this study, 516 preterm neonates’ data were included in the analysis. Two-third (66.86%) of the mothers were resided out of Gondar town. About one-fifth 417 (80.81%) of mothers were in the age range of 20–34 years with mean age of 26.52 years (Table 1).

**Table 1:** Socio-demographic and obstetric characteristics of mothers of preterm neonates admitted in NICU at University of Gondar comprehensive specialized Hospital from January 2016 to March 2018 (n = 516)

Characteristics	Frequency	Percent
Maternal residence		
Gondar town	171	33.14
Out of Gondar town	345	66.86
Age of women in years		
< 20	41	7.95
20–34	417	80.81
≥ 35	58	11.24
Had ANC in index pregnancy		
Yes	486	94.2
No	30	5.8
Parity (Number of births)		
I	230	44.57
II–IV	196	37.99
≥ V	90	17.44
Complication during index pregnancy		
Yes	120	23.26
No	396	76.74
Previous bad obstetrics history		
Yes	80	15.5
No	436	84.5
Type of pregnancy		
Singleton	331	64.15
Multiple	185	35.85
Onset of labor		
Elective caesarean section	56	10.85
Spontaneous	425	82.37
Induced	35	6.78
Place of birth		
Home	23	4.50
Health center	120	23.25
Hospital	373	72.25
Mode of delivery		
Spontaneous vaginal delivery	384	74.42
Caesarean section	117	22.67
Instrument assisted delivery	15	2.91
Duration of labour in hours (n = 460)		
< 4	52	11.30
4–18	354	77.00
> 18	54	11.70

#### Characteristics of the preterm neonates

Among 516 preterm neonates, 303 (58.73%) were males and 109 (21.12%) were small for gestational age. About one-sixth 82 (15.89%) had a body temperature of greater or equal to 36 °C measured within 1 h of admission and 371 (71.9%) were heated under radiant warmer. (Table 2).

**Table 2:** Characteristics of preterm neonates admitted in NICU at University of Gondar comprehensive specialized Hospital from January 2016 to March 2018 (n = 516)

Characteristics	Frequency	Percent
Sex of the neonate		
Male	303	58.73
Female	213	41.27
Gestational age (weeks)		
< 32	107	20.74
32–35	269	52.13
35–37	140	27.13
Weight for gestational age at birth		
Small	109	21.12
Appropriate	407	78.88
Newborn cry immediately at birth		
Yes	385	74.61
No	131	25.39
Bag and mask resuscitation at birth		
Yes	209	40.5
No	307	59.5
Newborns temperature with in 1 h of admission		
≤ 32	15	2.91
32.1–34	151	29.26
34.1–35	158	30.62
35.1–36	110	21.32
≥ 36	82	15.89
Peri-natal asphyxia diagnosed at birth		
Yes	137	26.55
No	379	73.45
Newborn diagnosed with respiratory distress		
Yes	142	27.52
No	374	72.48
Hypothermia diagnosed at admission		
Yes	426	82.56
No	90	17.44
Hypoglycemia diagnosed at admission		
Yes	112	21.71
No	404	78.29
Jaundice		
Yes	127	24.61
No	389	75.39
Newborn diagnosed with clinical sepsis		
Yes	401	77.71
No	115	22.29
Neonate received photo therapy		
Yes	143	27.71
No	373	72.29
Neonate received continuous positive airway pressure		
Yes	287	55.62
No	229	44.38
Newborn received kangaroo mother care		
Yes	68	13.18
No	448	86.82
Newborn heated with radiant warmer		
Yes	371	71.90
No	145	28.10

#### Proportion of preterm neonatal death

This finding showed that 149 (28.8%) with 95% CI; (25.1, 32.9) neonates died. From this 17 (11.4%) of them were died within the first 24 h of life and 127 (85.23%) died in the first 7 days of life (early neonatal death). The causes of death were multifactorial, not single. However, the leading causes were PNA (31%), HMD (26%), and cardiorespiratory arrest due to apnea (17%).

#### Associated factors of proportions of death for preterm neonates

Univariate and multivariable logistic regression was used to identify associated factors of death for preterm neonates admitted in the NICU.

Findings from bivariate analysis showed that complications during index pregnancy, previous bad obstetric history, neonatal respiratory distress, gestational age, small for gestational age, low APGAR score at birth, HMD, PNA, jaundice, receiving KMC, hypoglycemia, hypothermia, and temperature within 1 h of admission in °C were significantly associated with death of preterm neonates.

However, in the multi-variable analysis complications during the index pregnancy, neonatal respiratory distress, gestational age, small for gestational age, low APGAR score at birth, HMD, jaundice, receiving KMC, and hypoglycemia remained statistically significant factors.

The odds of death among preterm neonates delivered from mothers having complication during index pregnancy was 1.92 times higher as compared to their counterparts [AOR = 1.92; 95% CI (1.09, 3.38)].

The odds of death for preterm neonates who had < APGAR score at birth was 2.4 times higher than those who had > APGAR score at birth [AOR = 2.39; 95% CI (1.34, 4.27)].

Providing KMC for all preterm neonates reduce the odds of death by 87% as compared to not provided KMC [AOR = 0.13; 95% CI (0.05, 0.35)] (Table 3).

**Table 3:** Factors associated with proportion of death among preterm neonates admitted in NICU at University of Gondar specialized referral Hospital from January 2016 to March 2018 (n = 516)

Predictor variables	COR (95% CI)	AOR (95% CI)
Residence of the mother		
Gondar Town	1	1
Out of Gondar Town	1.66 (1.08, 2.54)	1.17 (0.67, 2.01)
Complication during index pregnancy		
No	*1*	*1*
Yes	*1.95 (1.27, 2.99)*	1.92 (1.09, 3.38)*
Previous bad obstetrics history		
No	1	1
Yes	1.82 (1.11, 2.98)	1.86 (0.95, 3.63)
Gestational age	*0.64 (0.57, 0.71)*	0.78 (0.69, 0.91)**
Weight for gestational age at birth		
Small	*2.05 (1.32, 3.18)*	2.42 (1.33, 4.38)*
Appropriate	*1*	*1*
APGAR score at birth		
≥ 7	*1*	*1*
< 7	*3.53 (2.32, 5.37)*	2.39 (1.34, 4.27)*
Neonates diagnosed for HMD		
No	*1*	*1*
Yes	*8.04 (4.98, 12.98)*	5.15 (2.83, 9.36)**
Neonatal hypothermia at admission		
No	1	1
Yes	2.10 (1.18, 3.74)	1.06 (0.46, 2.47)
Neonatal respiratory distress at admission		
No	*1*	*1*
Yes	*2.56 (1. 70, 3.85)*	1.93 (1.13, 3.31)*
Clinically diagnosed PNA		
No	1	1
Yes	2.65 (1.76, 4.01)	1.38 (0.77, 2.47)
Neonate diagnosed with jaundice		
No	*1*	*1*
Yes	*1.82 (1.19, 2.77)*	3.39 (1.90, 6.05)**
Neonate received KMC		
No	*1*	*1*
Yes	*0.25 (0.11, 0.55)*	0.13 (0.05, 0.35)**
Neonate diagnosed with hypoglycemia		
No	*1*	*1*
Yes	*2.48 (1.61, 3.84)*	3.86 (2.12, 7.06)*
Neonatal temperature measured within 1 h of admission in °C	0.69 (0.59, 0.80)	0.83 (0.67, 1.02)

* P-value < 0.05, ** P-value < 0.001

### Discussion

The proportion of death among preterm neonates admitted in University of Gondar Comprehensive Specialized Hospital NICU was 28.8% [95% CI (25.1, 32.9)]. The causes of death weren’t single problem rather combination of problems lead to death and the major once were PNA, HMD, jaundice, clinical sepsis and cardiorespiratory arrest due to apnea. All the causes are preventable by improving timely health seeking behavior of the community, upgrading the quality of care provided in the hospitals and health centers to be safe, clean and well equipped with all the infrastructures. This finding is in line with studies conducted in a multi-country level analysis reported by WHO and UNICEF 29.3% [14] and in Kenya 29.6% [19]. However, this finding was higher than studies conducted in a multi-country analysis by the lead of saving the children 15% [13] Cameroon 15.7% [18] Jimma Ethiopia 18.2% [20] and northern rural Ethiopia 23.7% [21].

This might be due to the difference in the study setting and the study population which was most vulnerable preterm neonates only.

In contrast, this finding was lower than studies conducted in population-based study from low to middle-income countries 37.5% [15], Urban Pakistan 34% [5], Jordan 40% [16], Johannesburg South Africa 64% [17], Tigray region Northern Ethiopia 34% [22–24] and Jimma University Specialized Hospital, Ethiopia 34.9% [26].

This might be due to the time variation where neonatal mortality is decreasing, access of health care service was increased, the health seeking and utilization behavior of the community and accessibility of trained health care providers are comparatively increased.

This finding showed that a neonate delivered from mothers with Complication during index pregnancy increased the odds of death by 92% as compared with their counterpart [AOR = 1.92, 95% CI (1.09, 3.38)]. This finding was supported by findings in northern Ethiopia [24], Ethiopian, DHS data [28], the sub-urban hospitals of Cameroon [18] and Johannesburg Central Hospital [29]. This similarity might be due to the complications (PROM, PIH, and delivery complications) affecting the pregnancy to result in preterm labour and acquired infections leading to neonatal death.

In this study, as the gestational age increase in a week, the odds of death was decreased by 22% [AOR of 0.78; 95% CI (0.69, 0.91)]. This finding was in line with findings in Jimma University specialized hospital [26] and Addis Ababa St. Paul’s Hospital Millennium Medical College [25]. This was because, as gestational age increases fetal maturity will be maximized and risk of life-threatening complications associated with prematurity may decrease.

This study showed that a neonate with small for gestational age at birth was 2.42 times at higher risk of death compared to appropriate for gestational age [AOR = 2.42, 95% CI (1.33, 4.38)]. This was supported by a study conducted in Jimma Zone [20] Ethiopian, DHS data [27], and Johannesburg Central Hospital [29]. The possible reasons might be due to that if small for gestational age the occurrence of life-threatening complications which lead to death is high compared to appropriate for gestational age neonates.

The odds of death was increased by 2.4 times for a neonate with APGAR score < 7 at birth as compared to the counterpart with [AOR = 2.39, 95% CI (1.34, 4.27)]. This result was comparable with the findings in Addis Ababa St Paul’s Hospital Millennium Medical College [25], sub-urban hospitals of Cameroon [18], and Taubaté University Hos-pital, Brazil [31].

In the current study, the odds of preterm neonatal death among cases of HMD was 5 times higher compared to none cases [AOR = 5.15, 95% CI (2.83, 9.36)]. This finding was comparable with findings in Jimma University specialized hospital [26] and Johannesburg Central Hospital [29]. This might be HMD is a disease of prematurity affecting respiratory function leading to death in preterm neonates.

The odds of death for preterm neonates with respiratory distress was 1.9 times higher than their counterparts [AOR = 1.93, 95% CI (1.13, 3.31)]. This finding was in line with findings of Butajira District, South Central Ethiopia [6], and Jimma University specialized hospital [26]. Because it is life-threatening complication leading to hypoxic-ischemic encephalopathy.

Preterm neonates with jaundice had 3.4 times higher odds of death than their counterparts [AOR = 3.39, 95% CI (1.90, 6.05)]. This finding was in line with findings in Jimma University specialized hospital [26]. This might be due to that gastrointestinal immaturity, liver enzyme deficiency leading to excess production of bilirubin to result in brain toxicity and death.

Hypoglycemia was significantly associated with the odds of death for preterm neonates, [AOR = 3.86, 95% CI (2.12, 7.06)]. This might be because of luck of adipose fat tissue serving as a source of glucose to adapt the extrauterine life until they maintain through feeding.

This finding showed that the odds of death among preterm neonates received KMC was lowered by 87% [AHR = 0.13, 95% CI (0.05, 0.35)]. This might be KMC prevents hypothermia by reducing body surface area to the external environment and helps easily accessing breastfeed on demand.

### Conclusions

In this study the proportion of preterm neonatal death was high. Complications during the index pregnancy, respiratory distress, gestational age, small for gestational age, low APGAR score at birth, HMD, jaundice, receiving KMC, and hypoglycemia were found to be significant factors for preterm neonatal death. All responsible bodies should work on quality care at ANC to maximize maternal health conditions, access NICU with infrastructures and skilled manpower at health institutions and give special care for preterm to avoid complications due to preterm.

## Limitation

It was a cross-sectional study and may not show the cause and effect relationship. A secondary source of data may lack some important variables affecting the outcome variables.

## Abbreviations

ANC: antenatal care
AOR: adjusted odds ratio
APGAR: Appearance Pulse Grimace Activity Respiration
APH: antepartum hemorrhage
B.Sc.: Bachelors of Science
CI: confidence interval
COR: crude odd ratio
EDHS: Ethiopian Demographic and Health Survey
GA: gestational age
HIV: human immune-deficiency virus
HMD: hyaline membrane disease
HR: hazard ratio
IRB: Institutional Review Board
KMC: kangaroo mother care
NEC: necrotizing enterocolitis
NCPAP: nasal continuous positive air pressure
NICU: neonatal intensive care unit
NGOs: non-governmental organizations
NMR: neonatal mortality rate
OR: odds ratio
PIH: pregnancy induced hypertension
PNA: perinatal asphyxia
PROM: prelabour rupture of membrane
PT: preterm
RDS: respiratory distress syndrome
UN: United Nations
UNICEF: United Nations International Emergency Children Fund
WHO: World Health Organization
